# *Carlota*, a new genus of Agrypnini from the Valdivian Forests of Chile (Elateridae, Agrypninae, Agrypnini)

**DOI:** 10.3897/zookeys.417.7012

**Published:** 2014-06-19

**Authors:** Elizabeth T. Arias-Bohart

**Affiliations:** 1 Essig Museum of Entomology, University of California, 1101 Valley Life Sciences Building, Berkeley 94720, California, U.S.A.

**Keywords:** Chile, Coleoptera, Elateridae, Agrypninae, Agrypnini, new species, new genus

## Abstract

*Carlota* **gen. n.**, with one included species *C. coigue* **sp. n.**, is described and illustrated from the Valdivian forests of Chile. The relationships of this genus to other Agrypnini from Chile are discussed and generic key for Chilean Agrypninae genera is provided.

## Abstract (es)

Se describe e ilustra *Carlota* **gen. n.**, con una especie *C. coigue* **sp. n.**, de los bosques Valdivianos de Chile. Se discuten las relaciones de este género con otros Agrypnini de Chile y se proporciona una clave para éstos.

## Introduction

Up to now, the Chilean Elateridae includes 52 genera and 140 species (Arias-Bohart and Elgueta 2012). The canopy beetle faunas of *Nothofagus* spp., and Araucarian forests show at least 32 yet undescribed Elateridae taxa (Arias et al. 2008). From those representing unknown Elateridae collected by fogging I found several specimens of a new Agrypnini.

## Materials and methods

This study is based on the specimens from multiple collecting trips of the Essig Museum of Entomology, University of California, Berkeley (led by E. T. Arias-Bohart) and private Chilean collections. The type specimens and loan material are indicated in the text. Acronysms of institutions and private collections follow Arnett et al. (1993).

ANIC Australian Insect Collection, Canberra, Australia

ETA Elizabeth Arias-Bohart, (private collection), Berkeley, California, USA

FMNH Field Museum of Natural History, Chicago, Illinois, USA

IRScNB Collections Nationales Belges d’Insectes et d’Arachnides, Institut royal des Sciences Naturelles de Belgique, Brussels, Belgium

MNHN Museum National d’Histoire naturelle, Paris France

JEB Juan Enrique Barriga Tuñon, (private collection), Curicó, Chile

MNNC Colección Nacional de Insectos, Museo Nacional de Historia Natural, Santiago, Chile

SRC Sergio Riese, (private collection), Genova, Italy

Specimens from which the genitalia were removed were first relaxed in 10% KOH solution over 1 to 3 days.

For examination of the male genitalia, the last abdominal segments were removed and placed in water with a few drops of soap in a Petri dish and left overnight. Then genitalia were subsequently extracted and placed into a small vial with 90% alcohol, or glued on a card, or on a vial, and pinned under the specimen. Methods outlined by Becker (1958) were followed for examination of the female genitalia. After examination, female genitalia were placed in a small vial with glycerin and pinned under the specimen.

Measurements. Following measurements were made with the aid of a calibrated ocular micrometer as follows: total body length from the frontal margin to elytral apex; pronotal length and maximum width of the pronotum, when both sides are in focus, and elytral length and maximum width of the elytra, when both sides are in focus.

Terminology. Terms for adult morphology follows Platia (1994) and Calder (1996). Wing vein nomenclature follows that of Dolin (1975), Kukalová-Peck and Lawrence (1993, 2004).

Label Information. Places and names of the material studied are from the original spellings from recorded specimen labels. The following symbols are used in the recorded label information as follows:

/ indicating line separation within label, // indicating label separation. Juan Enrique Barriga’s collection labels include the following URL http://www.coleoptera-neotropical.org, which I have excluded from the label information.

Drawings were made using a camera lucida on a Leica MZ7 dissecting scope. Type material has been databased with a unique number indicated on the label information consisting of the acronym EMEC and the identification number. For example, the holotype of *Carlota coigue* sp. n., has the unique number EMEC10005989 followed by the repository in brackets. Type information of the described material is available online at essigdb.berkeley.edu.

## Taxonomy

### Carlota gen. n.

Taxon classificationAnimaliaColeopteraElateridae

http://zoobank.org/C20C8BB5-58D2-4477-A501-C81F9EE77C5C

#### Type species.

*Carlota coigue* sp. n., here designated.

#### Etymology.

The generic name Carlota (gender feminine) is dedicated to my mother Carlota Tobar Vega, who has always encouraged me in my study of nature and insects.

#### Diagnosis.

This genus differs from all other elaterid genera by the following characters: antennal grooves short, pronotum subquadrate; mesepisternum forming part of mesosternal cavity, mesosternal cavity shape oval; mesosternal posterior region pointed; mesocoxal distance about 4.6 times mesocoxal cavity. Wing venation with R cell short MP_3+4_ bent towards MP_1-2_ not branching towards MP4+CuA_1._

#### Description.

Body about 3.72–4.22 times as long as wide, sides subparallel from anterior pronotal sides towards elytral sides, slightly narrowing posteriorly towards elytral apices. Dorsal vestiture short dense, fine, with some erect short well distributed hairs.

Head declined at base, transverse, ratio of median length to greatest postocular width 0.24. Eyes medium sized, protuberant in both sexes, facetted, lacking interfacetal hairs. Supra-antennal ridges strong, fossa shallow. Frontoclypeal region flattened and frontally carinate. Labrum small, transverse, sclerotised, sinuate basally. Antennae in male with antennomeres 3–10 strongly serrate, antennomere 11 elongate, much longer than preceding ones; all antennomeres clothed with long and short semi-erect and erect gold hairs. Female antennae shorter than male antennae.

Prothorax subquadrate, about 0.70–0.90 times as long as greatest width. Sides almost straight or slightly expanded posteriorly, carinate and emarginate, not visible for their entire length viewed dorsally. Posterior angles short and stout, produced posterolateraly. Posterior edge with scutellar notch broad and sharply defined. Disc punctate, clothed with dense hairs. Prosternum more or less flat with deep punctures. Notosternal suture complete, straight for most of its length, open anteriorly; curved posteriorly. Prosternal process narrow near base, then gradually expanded posteriorly, following procoxae in lateral view, extending well behind procoxae. Hypomeron simple, with deep punctures. Procoxae subglobular.

Elytra dark brown or black, about 2.79–3.18 times as long at midline as greatest width and 4.09–4.90 times as long as pronotum. Humeri well developed; parallel-sided anterior 2/3rds, gradually converging posteriorly, apices rounded. Disc with 10 weakly defined puncture rows. Mesoventrite on same plane as metaventrite. Mesocoxae slightly projecting, mesocoxal cavities narrowly separated, open laterally to mesanepisternum. Metacoxae obliquely oriented, with plates not extending to lateral edges of coxae.

Hindwing about 2.83 times as long as wide. Apical field about 0.6 times as long as total wing length, with 2 lightly pigmented oblique linear sclerites. Radial cell well developed, elongate, 3.4 time as long as wide, with inner posterobasal angle forming a right angle. Cross-vein r_3_ moderately short, horizontal and arising distally from r_4_, which is mostly linear and complete. Base of RP very long, extending to wing base. R-M loop forming a narrowly acute angle; medial spur slightly curved. Medial field with five free veins. MP_3+4_ not branching in 2 veins (Figs 14, 16, 17).

Tarsomeres. 1–3 elongate, tarsomere 4 smaller than precedents. Pretarsal claws simple; empodium short, not extending between claws.

Female genitalia: bursa copulatrix globular, 1.51 mm in diameter, with one sclerotised internal structure comb-shape, with numerous spinules, mostly shorts and few longs (Fig. 21).

Aedeagus. Symmetrical, attached to parameres both dorsally and ventrally.

#### Distribution.

Chile provinces: Curicó, ñuble, Malleco, Cautín.

### Carlota coigue sp. n.

Taxon classificationAnimaliaColeopteraElateridae

http://zoobank.org/F69AB8E3-1F79-4CBE-AF19-B9FA7502C218

Figs 1 , 4 , 10 , 15 , 20 , 24–26

#### Etymology.

This species is named after the evergreen tree *coigüe* (vernacular name of *Nothofagus dombeyi*) is to be considered a noun in apposition.

#### Description.

**Figure 1.**
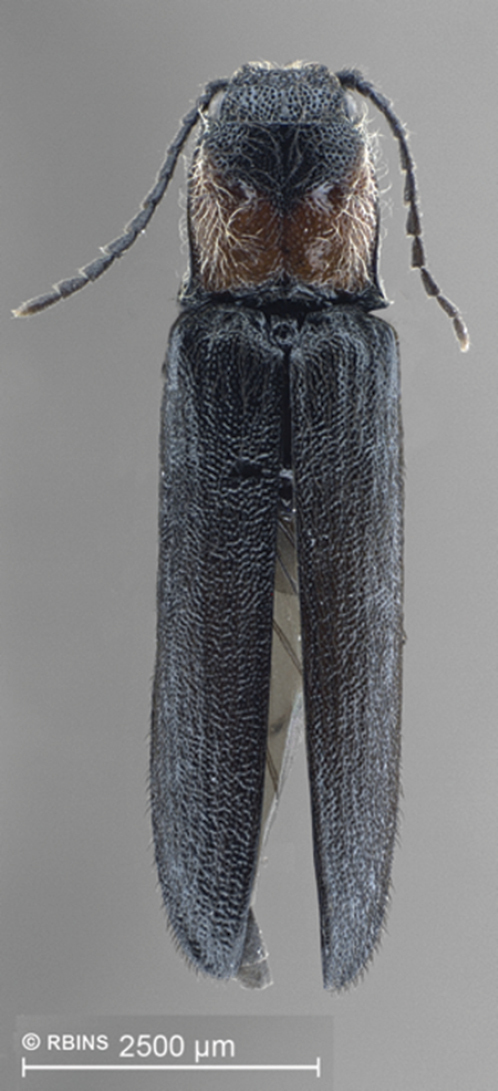
Adult of *Carlota coigue*.

Holotype ♂: Body brown; integument dull, body length 8.59 mm, width 2.16 mm (Fig. 1).

Head dark brown, deeply inserted into prothorax antenna same color as head. Antennomere 10 reaching apex of posterior pronotal angles, antennomere 3 smaller than antennomere 2, antennomere 5 through eighth similar in length, antennomere 11 about 1.6 times antennomere 10 (Fig. 24). Mandibles curved and acute (Fig. 25).

Prothorax anteriorly black and posteriorly with a reddish triangular area, with long gold semi-decumbent hairs, 1.36 times as long as wide. Punctate, punctures separated by more than one own diameter. Pronotal hypomeron base straight posteriorly.

Scutellum orange at middle. Elytra black or dark brown, 3.04 times as long as wide. Legs brown, vestiture black. Tarsomeres 2 and 4 more or less equal in length, tarsomere 4 only half as long as 1.

Male genitalia. Length 1.89 mm, and 0.35 mm wide. Parameres apex globose with a hook, with at least 3 strong setae (Fig. 26).

**Figures 2–7.**
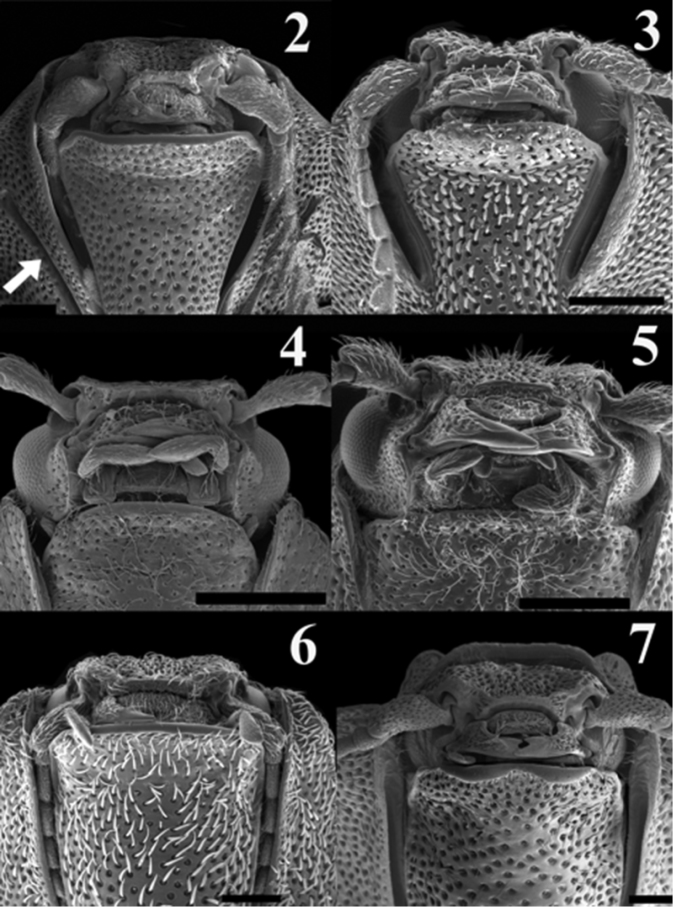
Scaning electron micrograph of frontal head of: *Acrocryptus ater* (**2**)*Agrypnus* sp. (Australia) (**3**) *Candanius* sp. (**4**) *Carlota coigue* (**5**)*Dilobitarsus laconoides* (**6**)*Lacon chilensis* (**7**). Scale bar = 1 mm.

**Figures 8–13.**
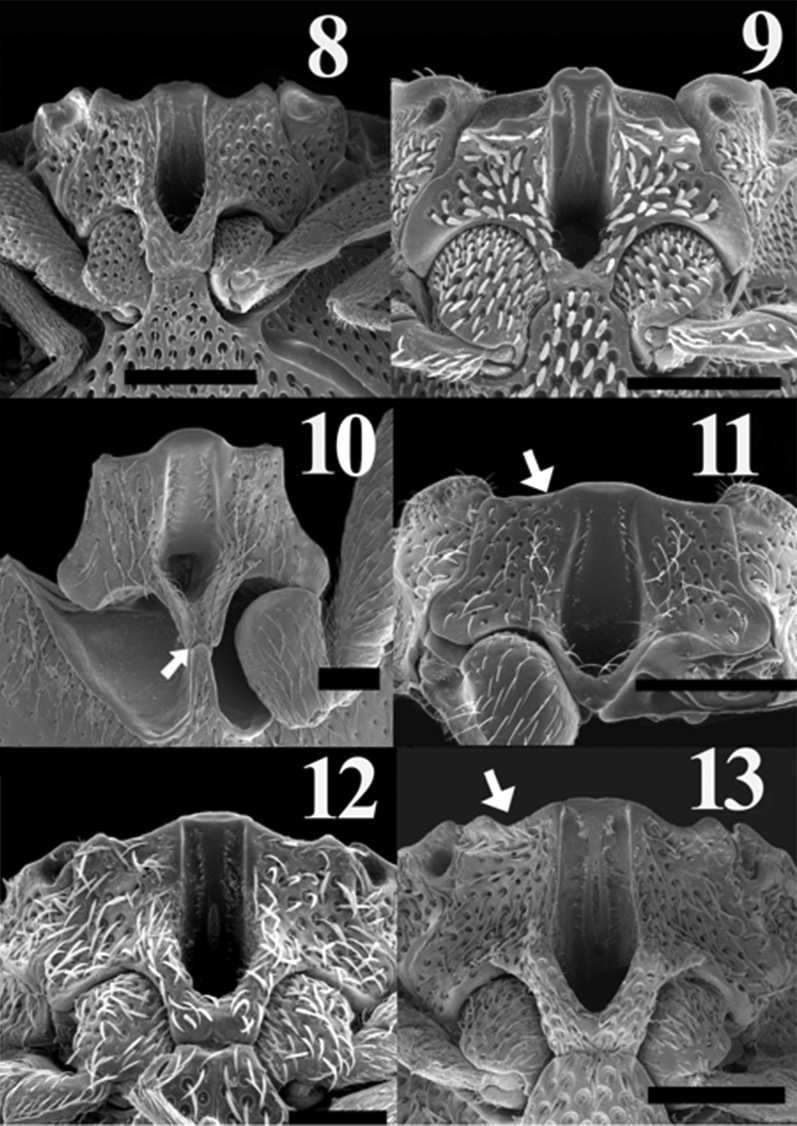
Scaning electron micrograph of mesoventral cavity of: *Acrocryptus ater* (**8**)*Agrypnus* sp. (Australia) (**9**) *Candanius* sp. (**10**) *Carlota coigue* (**11**)*Dilobitarsus laconoides* (**12**)*Lacon chilensis* (**13**). Scale bar = 1 mm.

**Figures 14–19.**
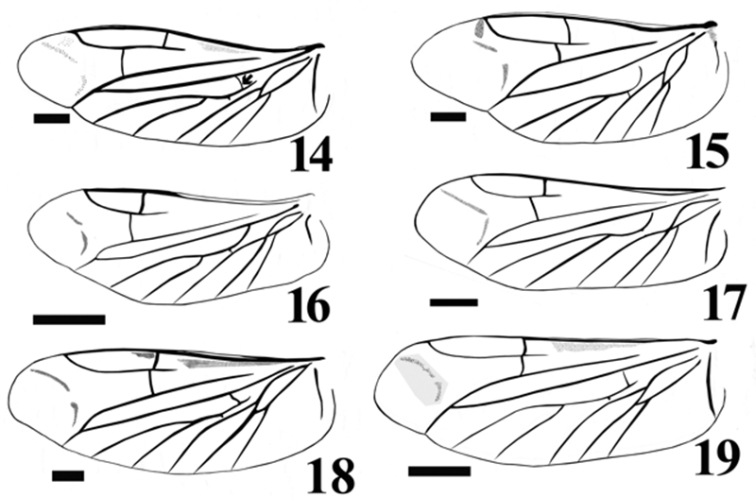
Wing venation illustration of: *Acrocryptus ater* **14** *Agrypnus* sp. (Australia) **15** *Candanius* sp. **16** *Carlota coigue* **17** *Dilobitarsus laconoides* **18** *Lacon chilensis* **19.** Scale bar = 1 mm.

**Figures 20–23.**
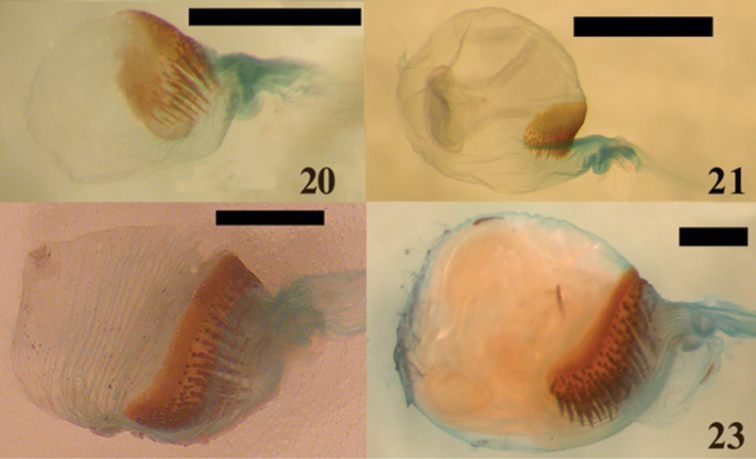
Female genitalia of *Candanius* sp. **20** *Carlota coigue* **21** *Dilobitarsus laconoides* **22** *Lacon chilensis* **23** Scale bar = 0.5 mm.

**Figures 24–26.**
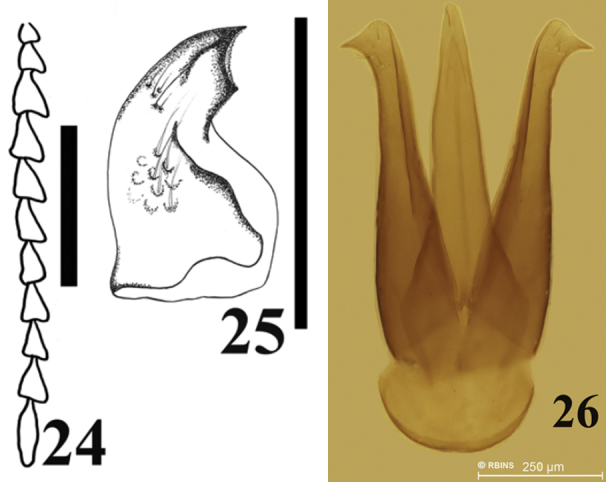
Antennomeres, mandible and male genitalia of *Carlota coigue*. Antennomeres (**24**) mandible (**25**) male genitalia (**26**). Scale bar = 0.5 mm.

#### Distribution.

Chile provinces: Curicó, ñuble, Malleco, Cautín.

#### Type material.

HOLOTYPE. On a card, ♂genitalia // 15-CHILE IX Region / Flor del Lago Ranch Villarrica / 39°12'378"/ 78°08'182"312m // 12.XII.2003 /Canopy Fogging 60cc/l / Arias et al UCB // HOLOTYPE /*Carlota coigue* ♂/ E. Arias-Bohart 2013 //EMEC10005989// [MNNC]

PARATYPES. On a card //CHILE- ÑUBLE Shangri-lá / 6-11-12, 1998 / col. J. Mondaca // Paratype/ *Carlota coigue* ♂ / E. Arias-Bohart 2013 //EMEC10006002// [MNNC]

On a card // abdomen //15-CHILE IX Region / Flor del Lago Ranch Villarrica / 39°12'378” / 78°08'182”312m / 12.XII.2003 / Canopy Fogging 60cc/l / Arias et al UCB //

Paratype/ *Carlota coigue* ♂/ E. Arias-Bohart 2013 //EMEC10005991// [ANIC]

On a card // female genitalia on a vial //15-CHILE IX Region /Flor del Lago Ranch Villarrica/ 39°12'378"/ 78°08'182"/312m / 12.XII.2003 / Canopy Fogging 60cc/l / Arias et al UCB //Paratype/ *Carlota coigue* ♀/ E. Arias-Bohart 2013 //EMEC10005990// [ETA]

On a card //Shangrila /VIII Region/30-10-1988 /Elizabeth Arias // Paratype /*Carlota coigue* ♂ /E. Arias-Bohart 2013 //EMEC10005993// [ETA]

On a pin //Chile Marimenuco /Lonquimay / 10-15.XII.1986 / Coll. L.E. Peña // Paratype /*Carlota coigue* ♂ / E. Arias-Bohart 2013 //EMEC10005996// [ETA]

On a card //CHILE: Cautín P.R.: P.N./ Conguillío, 1.5 Km East/Laguna Captrén, guard sta.1365m, / 38°38'7"S, 71°41.37'W, // 23.xii.1996–5.ii.1997 //*Nothofagus dombeyi*/ deciduous spp., /*Araucaria*, with /*Chusquea* understory/ FMHD #96-229, flight/ intercept trap. A.New- / ton & M.Thayer 977/Field Mus. NAT. HIST. //Paratype /*Carlota coigue* ♂ / E. Arias-Bohart 2013 //EMEC10005994// [FMNH]

On a point //Chile, prov. Curicó, 15/ km. E. Potrero Grande, / Camino El Relvo, 19/ Leg. JE. Barriga T,/ S/N. alpina, N. obliqua/ 36011.14 S700 56.1W // Colección J. E. Barriga //CHILE 163778 // Paratype/ *Carlota coigue* ♂// E. Arias-Bohart 2013 //EMEC10005998// [IRScBN]

On a card // male genitalia // Chile Talca 1300 m. /Altos de Vilches/ 26.I.69 Valencia // Ex-colección / Jorge Valencia / JVCC / Chile 003660 // Colección JEBC / Juan Enrique Barriga-Tuñon / Chile 0204053 // Paratype/ *Carlota coigue* ♂ // E. Arias-Bohart 2013 //EMEC10005999// [JEB]

On a card // on a card male genitalia // Chile Arauco / Pichinahuel / 15.I.59 / G. Barria // Ex. Colección / Jorge Valencia /JVCC /Chile 003152 /Valencia //Ex-colección / Jorge Valencia /JVCC/ Chile 003660 //Colección JEBC /Juan Enrique/Barriga-Tuñon /Chile 0204684 // Paratype/ *Carlota coigue* ♂ //E. Arias-Bohart 2013 //EMEC100060000// [JEB]

On a point // CHILE prov. Ñuble/ Shangri-lá, 1490 mt 36°52'34"S, 71°28'3"W, 7 / dic 2008. Fogging Lenga (Nothofagus pumilio) / leg J. E. Barriga-Tuñon // Colección / JE Barriga-Tuñon / Chile 122722 // Paratype/*Carlota coigue* ♂ / E. Arias-Bohart 2013 // EMEC10006001 // [JEB]

On a card // male genitalia // Chile, prov. Curicó, 15/ km. E. Potrero Grande, Puente Morongos, 25/ nov 2003, fogging a/ Nothofagus dombeyi/ Nothofagus dombeyi/ S36°12'58.1"/ W70°58'37.4/ leg. J. E. Barriga// Colección J. E. Barriga // CHILE 148098 //Paratype / *Carlota coigue* ♂ // E. Arias-Bohart 2013 //EMEC10005997// [MNHN]

On a card, specimen & male genitalia //15- CHILE IX Region /Flor del Lago Ranch Villarrica /39°12'378"/78°08'182"312m / 12.XII.2003 / Canopy Fogging 60cc/l / Arias et al UCB // Paratype /*Carlota coigue* ♂ / E. Arias-Bohart 2013 //EMEC10005992// [SRC]

### Key to separate Chilean genera of Agrypnini

**Table:** 

1	Antennal groove short, less than half of length of pronotosternal suture; articulate surfaces of mesosternite not angulate (Fig. 11, white arrow); mesocoxal distance more than 4 times the length of the mesocoxal diameter	2
–	Antennal groove more than half of length of pronotosternal suture; articulate surfaces of mesosternite angulate (Fig. 13, white arrow); mesocoxal distance less than 4 times the length of the mesocoxal diameter	3
2	Pronotum elongate, lacking distinctive depressions; prosternal process following procoxae; posterior region of mesosternum excavate (Fig. 10, white arrow)	*Candanius* Hayek
–	Pronotum subquadrate, with distinctive depressions; prosternal process straight; posterior region of mesosternum pointed (Fig. 11)	*Carlota* gen. n.
3	Mesosternal cavity length less than three times its width (Fig. 13); tarsomeres lacking ventral lobes	*Lacon* Laporte
–	Mesosternal cavity length more than three times its width; tarsomeres with ventral lobe	4
4	Antennomeres flabellate (Fig. 2); grooves for anterior, middles, and hind tarsomeres present on propleura, metasternum and abdominal sternite respectively (Fig. 8); mesosternum anteriorly with a small notch	*Acrocryptus* Candéze
–	Antennomeres serrate (Fig. 6); lacking associated grooves for anterior, middles, and hind tarsomeres; mesosternum anteriorly without a small notch (Fig. 12)	*Dilobitarsus* Latreille

## Discussion

The subfamily Agrypninae is considered one the most ancient subfamilies in Elateridae (Gurjeva 1969, Dolin 1978). However, even though several workers have treated this subfamily, its placement within the family is still insufficiently settled since most of the characters used to establish genus-level taxa in more advanced subfamilies of Elateridae show high variability within the Agrypninae at both the generic and specific level (Prosvirov and Savitsky 2011).

Hayek (1973) studied the characters to separate the genera of Agrypninae based on the structure of the middle coxal cavity, and was followed by Nakane and Kishii (1956) who also included the size and shape of the second and third antennal segment. Later, Gurjeva (1974) demonstrated the importance of the characters of the prothorax and metathorax, as well as those characters found on the bursa copulatrix as important in establishing natural supraspecific and suprageneric groups. Also, Gurjeva (1974) indicated that the rearward extent of the lateral lobes of the mesosterinite was a subfamily character. I also found important the shape of the lateral lobes of the mesosternite, the shape and depth of the groove of the mesothoracic cavity important.

In Chile the tribe Agrypnini has 4 genera *Acrocryptus* Candèze, 1874, *Candanius* Hayek, 1874, *Dilobitarsus* Latreille, 1874, and *Lacon* Laporte, 1838 distributed in 8 species as follows: *Acrocryptus ater* (Philippi, 1873), *Candanius gracillimus* (Candèze, 1889), *Dilobitarsus crux* (Philippi & Philippi, 1860), *Dilobitarsus laconoides* (Fleutiaux, 1907), *Dilobitarsus sulcicollis* (Solier, 1851), *Dilobitarsus vitticollis* (Fairmaire & Germain, 1860), *Lacon chilensis* (Solier, 1851) and *Lacon fairmairei* (Candèze, 1881).

*Carlota* appears to be closely related to the genus *Candanius* because they share the following characters: short antennal grooves, not angulate articulate surfaces of mesososternite (Fig. 11, white arrow). Mesocoxal distances more than four times the mesocoxal diameter, and wing vein MP_3+4_, curves towards major vein MP_1+2_, without branching in two short veins (Figs 16, 17).

*Carlota* differs from *Candanius* by the following (contrasting characters for *Candanius* in parentheses): posterior prosternal lobe truncate (Fig. 5) (posterior prosternal lobe curved Fig. 4), posterior region of mesosternum pointed (between mesocoxae Fig. 11) (posterior region of mesosternum excavate (between mesocoxae) Fig. 10), wing with two elongate plates (Fig. 17) (wing with two short plates, Fig. 16), prosternal spine straight (prosternal spine follows procoxae), bursa copulatrix with a sclerotised structure about 0.37 times the bursa copulatrix diameter, (Fig. 21) (bursa copulatrix with a sclerotised structure about 0.68 times the bursa copulatrix diameter, Fig. 20).

*Carlota* differs from *Acrocryptus* by the following (contrasting characters for *Acrocryptus* in parentheses): short antennal grooves not well developed for the reception of the antennae (Fig. 5) (long antennal grooves for the reception of the antennae extending beyond the anterior half of the prosternopleural suture, Fig. 2), lacks grooves on propleura, metasternum and abdominal sternite for anterior, middles, and hind tarsomeres (posses grooves on propleura, metasternum and abdominal sternite for anterior, middles, and hind tarsomeres, Fig. 8), third and fourth tarsal segments without lobes (third and fourth tarsal segments with lobes).

*Carlota* differs from *Dilobitarsus* by the following (contrasting characters for *Dilobitarsus* in parentheses): short antennal grooves not extending beyond the anterior half of the prosternopleural suture, (Fig. 5) (antennal grooves extending beyond the anterior half of the prosternopleural suture, Fig. 6), mesosternal cavity sides anterior half region not parallel, (Fig. 11) (mesosternal cavity sides anterior half region parallel, (Fig. 12), third and fourth tarsal segments without lobes (third and fourth tarsal segments with lobes), wing R cell less than 2 times its width, (Fig. 17) (wing R cell more than 4 times its width, Fig. 18).

*Carlota* differs from *Lacon* by the following (contrasting characters for *Lacon* in parentheses): short antennal grooves not extending beyond the anterior half of the prosternopleural suture, (Fig. 5) (antennal grooves extending beyond the anterior half of the prosternopleural suture, Fig. 7), mesocoxal distance more than 4 times mesocoxal diameter, (Fig. 11) (mesocoxal distance less than 3 times mesocoxal diameter, Fig. 13), anterior region of mesosternum lacking a small notch, (Fig. 11) (anterior region of mesosternum with a small notch, Fig. 13), articulate lobes of mesosternum not angulate, (Fig. 11, white arrow) (articulate lobes of mesosternum angulate, Fig. 13).

*Carlota* most closely approaches the genus *Agrypnus* (studied *Agrypnus* sp. from Australia) which does not occur in Chile. It differs from *Agrypnus* by the following (contrasting characters for *Agrypnus* in parantheses): short antennal grooves, not extending beyond anterior half of the prosternopleural suture, (Fig. 5) (deep antennal grooves extending beyond anterior half of the prosternopleural suture, Fig. 3), anterior region of mesosternum not notched, (Fig. 11) (anterior region of mesosternum notched, Fig. 9), mesosternal cavity length less than 3 times its width, (Fig. 11) (mesosternal cavity length more than 3 times its width, Fig. 9), mesosternal cavity sides not parallel, (Fig. 11) (mesosternal cavity sides parallel, Fig. 9).

Based of the appearance of the prothorax, Hayek (1973) concluded that the retention of *Lacon* and *Dilobitarsus* could not be justified. However, I consider these two genera valid based on the following: *Dilobitarsus* differs from *Lacon* by the following (contrasting characters for *Lacon* in parentheses): walls of anterior half of the mesosternal cavity sides mostly parallel (walls of anterior half of the mesosternal cavity sides divergent), posterior region of the mesosternal cavity subquadrate, (Fig. 12) (posterior region of the mesosternal cavity V-shaped, Fig. 13), tarsomeres with lobes (tarsomeres without lobes), bursa copulatrix subglobular (bursa copulatrix globular), sclerotised structure of bursa copulatrix about 0.4-0.5 times the diameter of bursa copulatrix, (Fig. 22) (sclerotised structure of bursa copulatrix about 0.3 times the diameter of bursa copulatrix, Fig. 23).

Discovery of the larvae of the above genera will likely help clarify the systematic position of the tribe Agrypnini and its members.

## Supplementary Material

XML Treatment for Carlota

XML Treatment for Carlota coigue
